# Single Stage Endovascular Treatment of a Type 2 Abernethy Malformation: Successful Nonsurgical Outcome in a Case Report

**DOI:** 10.1155/2015/491867

**Published:** 2015-12-06

**Authors:** Carl Kraus, Vladimir Sheynzon, Robert Hanna, Joshua Weintraub

**Affiliations:** Department of Radiology, Columbia University Medical Center, New York Presbyterian Hospital, New York, NY 10032, USA

## Abstract

Abernethy malformations are a rare collection of congenital hepatic portosystemic shunts. Our 19-year-old patient is with a type 2 Abernethy malformation elected permanent shunt closure following worsening dyspnea. This report details a single stage endovascular technique wherein shunt closure was achieved immediately by placement of an aortic endograft. At 5-month follow-up, the patient reported decreased shortness of breath. Furthermore, ultrasound investigation demonstrated a patent portal vein and right heart catheterization 6 months after procedure revealed decreased pulmonary hypertension relative to preprocedure values. This one step method may serve as an alternative treatment strategy to multistage endovascular closure techniques of type 2 Abernethy malformations.

## 1. Background

The Abernethy malformations, which include types of congenital portosystemic shunts (CPSS), are rare vascular anomalies of the portal venous system [1]. As implied by the name, CPSS involve diversion of portal venous blood into the systemic venous circulation, thus bypassing the liver. CPSS are classified into two types: type 1 CPSS are characterized by complete shunting and absence of a portal vein, while type 2 CPSS exhibit partial shunting and a hypoplastic portal vein [2].

CPSS may manifest with a variety of physiologic derangements such as hepatic encephalopathy, pulmonary hypertension, and hypoxemia. Additionally, these shunts have been associated with neoplastic liver disease such as hepatocellular carcinoma and hepatoblastoma [3, 4]. Due to the significant morbidity that accompanies Abernethy malformations, corrective intervention may become necessary.

The treatment of these vascular anomalies has been dictated by the type of malformation. Type 1 malformations, with an absent portal vein, are definitively treated by liver transplantation [5]. Type 2 malformations have been successfully treated with either surgical ligation or endovascular shunt occlusion [6, 7]. Recently there have been reports detailing a variety of multistage endovascular occlusion techniques in the treatment of type 2 malformations [8, 9]. Herein, we present a case in which a type 2 Abernethy malformation was successfully occluded in a single stage placement of an aortic endograft within the inferior vena cava (IVC). To our knowledge, this is the first report of a successful immediate, single stage CPSS closure utilizing this method of vessel occlusion.

## 2. Case

A 19-year-old male with a past medical history of asthma, peptic ulcer disease, and pulmonary hypertension secondary to type 2 CPSS presented with a two-month history of worsening shortness of breath and exertional dyspnea. In 2005, he had been diagnosed with a type 2 Abernethy malformation during workup of acute asthma exacerbation. In 2010, he was diagnosed with pulmonary hypertension after presenting with multiple episodes of hemoptysis. His home medications at the time of the procedure included tadalafil (Adcirca) 40 mg daily, esomeprazole 20 mg daily, and inhaled albuterol every four hours as needed for dyspnea. The patient's past surgical history, family history, and social history were noncontributory. Magnetic resonance imaging demonstrated an aneurysmally dilated intrahepatic portal venous shunt communicating with the inferior portion of the intrahepatic inferior vena cava. The maximum diameter of the shunt measured 2.9 cm, with the neck of the shunt measuring 0.9 cm. Additionally, preprocedural transjugular liver biopsy confirmed patent intralobular hepatic portal veins accompanied by mild centrilobular dilation and no evidence of fibrosis. Due to worsening respiratory symptoms refractory to medical management, the patient elected to undergo endovascular closure of the CPSS.

**Figure 1:**
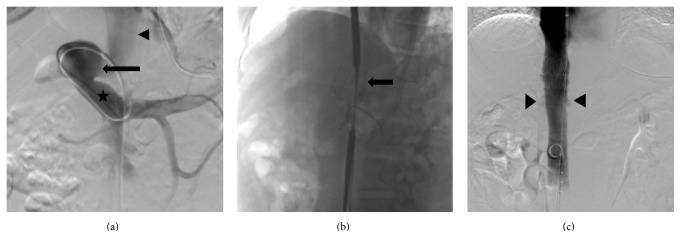
(a) Digital subtraction angiography of a type 2 Abernethy malformation. Injection of contrast into the splenic vein revealed marked flow of contrast into a patent portal vein (star), the portosystemic shunt (horizontal arrow), and the IVC (arrowhead). (b) Fluoroscopic placement of aortic endograft within the IVC. Introduction of the aortic endograft (arrow) into the IVC occluded the communication between the portosystemic shunt and systemic venous system. Care was taken not to occlude the renal or hepatic veins. (c) Postprocedure cavagram. Digital subtraction venography demonstrates a patent inferior vena cava after endograft placement at T12-L1. The inferior aspect of the graft can be visualized in satisfactory position superior to the renal vein inflow bilaterally (arrowheads).

For the procedure, intravascular access was gained via the right internal jugular vein (IJ) with a 5 F micropuncture kit. After wire introduction and serial dilations, a 5 F C2 cobra catheter was introduced over an 0.035 Amplatz wire into the CPSS malformation via the IVC, allowing for shunt visualization (Figure 1(a)). In order to assess the patency of the portal system, transjugular pressure measurements were obtained prior to the procedure. Pressure measured via the IVC shunt was as follows: portal vein pressure was 8 mmHg, wedged hepatic pressure was 16 mmHg, and free hepatic pressure was 12 mmHg. Next, femoral vein access was gained in a similar fashion to IJ access, and the renal vein was entered using a 5 Fr cannula. The renal vein location was noted so as to avoid its occlusion during placement of the aortic endograft. Following serial dilations at the internal jugular vein, a 20 F sheath was introduced, and a 32 mm × 39 mm Zenith Flex stent graft was deployed (Figure 1(b)), excluding the connection of the shunt to the IVC. The graft was placed so as not to occlude the renal veins inferiorly or the hepatic veins superiorly. A venacavogram was performed after balloon dilatation of the stent (Figure 1(c)) demonstrating exclusion of the portal-caval shunt.

The patient's postprocedure course was complicated by umbilical abdominal pain, urinary retention, and hypoxia. These symptoms resolved on postprocedure day two. Portal pressure measurements on postoperative day two were 12 mmHg hepatic wedge and 7 mmHg free hepatic. The portal venous system could not be measured directly due to closure of the shunt. Ultrasound Doppler flow studies demonstrated adequate flow within the main portal vein. The patient was discharged home in stable condition on postprocedure day three.

At five-month follow-up, the patient reported decreased exertional dyspnea and improved quality of life. Ultrasound Doppler flow studies at that time demonstrated a patent main portal vein. At six-month follow-up, right heart catheterization was performed. Pre- and postprocedure right heart catheterization showed systolic pulmonary artery and right atrial pressure decreased from 62 mmHg and 13 mmHg to 49 mmHg and 9 mmHg, respectively.

## 3. Discussion

The first Abernethy malformation was described by John Abernethy in 1793 upon autopsy of a deceased 10-month-old female. Since his hallmark case, further classification systems have been devised to differentiate the variety of CPSS based on the pathway of shunting [2, 6]. The classification of the CPSS guides the method of treatment, wherein type 1 necessitates liver transplantation while type 2 has been shown to be amenable to endovascular occlusion [6, 7].

Successful repair of type 2 malformations hinges on the ability of the hypoplastic portal system to accommodate increased blood flow. Acute closure of the portosystemic shunt in the presence of a profoundly hypoplastic portal system can result in severe mesenteric congestion [7]. However, multiple studies have shown that, in the setting of type 2 Abernethy malformations, portal veins have the capacity to develop de novo or reexpand following shunt closure [6–9].

A report by Kuo et al. describes a staged Abernethy repair that was achieved by placing an occlusive, central venous covered stent [8]. The stent placement was preceded by TIPS placement since liver biopsy had demonstrated a paucity of intact portal venous structures. Gradual closure of the TIPS corresponded with development of an increased number of patent portal veins within the liver. A similar study by Bruckheimer et al. utilized sequentially placed stents of smaller diameters within the shunt that were progressively closed over time. Both of these reports demonstrate the plasticity of the portal venous system following multiple stages of flow restriction.

Our present case report describes a successful single stage repair utilizing a central covered venous stent. Staged closure or TIPS placement was not performed because preprocedure liver biopsy demonstrated intact intralobular portal veins. Avoidance of multistage procedures reduces radiation exposure which is of particular concern in pediatric patients. The procedure outcome was monitored with serial Doppler ultrasound exams of the liver and portal vein which demonstrated patency 5 months after the procedure. Furthermore, pre- and postprocedure transjugular pressure measurements revealed a decrease in hepatic wedge pressure. This finding suggests an intact, low pressure portal system following percutaneous intervention. Right heart catheterization demonstrated a decrease in pulmonary artery pressure of 13 mmHg, which correlated with improvement of clinical symptoms. Together, these measurements illustrate the effectiveness of this single stage closure technique.

Although the technique used in this instance avoided the need for multiple procedures, certain preprocedure considerations are essential to safely perform this endovascular approach. Preprocedure biopsy must demonstrate an intact portal venous system. Preprocedure biopsy which demonstrates an absence of portal venous system would be consistent with a type 1 Abernethy malformation and therefore would be solely amenable to liver transplantation. In order to perform the single staged closure of a type 2 Abernethy malformation, histologic demonstration of an intact portal venous system is essential.

Special attention should also be given to the location of the renal veins and the hepatic veins before occlusion of the aberrant shunt. In the case described in this report, the shunt was located superior to the renal veins but inferior to the hepatic veins. This anatomy was observed on MRI prior to the procedure and was confirmed in real time with angiography prior to the placement of the occluding stent (Figure 1(c)). Strategies to avoid occlusion of the native renal and hepatic veins include differential placement as well as modification of the vertical height of the occlusive stent.

While a covered stent was chosen as the occluding device, an alternative option would be placement of an Amplatzer plug. Amplatzer plug placement would be a less rigid device than the covered stent used in this case and thus possibly would result in less postoperative pain. However, use of such a device also carries a potential risk for dislodgement and embolization to the right heart, and thus proper sizing would be essential prior to placement.

In summary, this report details an effective, single stage endovascular technique for the occlusion of a congenital portosystemic shunt. Acute closure of CPSS can be achieved if there is histologic evidence of an intact portal venous system. Minimally invasive management of CPSS in a single stage procedure provides a safe and effective intervention for type 2 Abernethy malformations.

## Abbreviations

CPSS: Congenital portosystemic shunts
IVC: Inferior vena cava.
